# ‘Some of Them Were Created by People Who Have Never Had a Stroke’: Understanding Desired Physical and Occupational Therapy Research Study Features for People With Chronic Stroke

**DOI:** 10.1111/hex.70918

**Published:** 2026-10-08

**Authors:** Sarah M. Schwab‐Farrell, Jasmine Ficke, Daria Pressler, Kelly Farrell, Becca Rose, Valerie Miller

**Affiliations:** ^1^ Department of Rehabilitation Exercise, & Nutrition Sciences, University of Cincinnati Cincinnati OH USA; ^2^ Division of Occupational Therapy & Physical Therapy, Cincinnati Children's Hospital Medical Center Cincinnati OH USA

**Keywords:** participatory research, qualitative, rehabilitation, stroke

## Abstract

**Background:**

People with stroke have historically had limited involvement in physical and occupational therapy research studies. Scientists and funders, rather than those most affected by the outcomes of research, largely make decisions about what studies should focus on. When research is not informed by the lived experiences of people with disability, researchers may ask the wrong questions or make incorrect assumptions about disability.

**Objective:**

The purpose of this study was to identify desired features and characteristics for physical and occupational therapy research studies, from the perspective of people with chronic stroke.

**Methods:**

We completed four iterative focus group discussions with a small group of adults with chronic stroke. The group was purposively sampled to ensure a range of prior stroke research experiences and stroke‐related impairments. Thematic analysis was conducted on discussion content to identify desired study features.

**Results:**

Four major desired study features were constructed: (1) Desire for relationships; (2) Understand the purpose; (3) Capability‐oriented; and (4) Focused on individual participation needs. Participants expressed a desire for physical and occupational therapy studies to focus on what they can do (rather than what they cannot do) while also embracing the heterogeneity of stroke presentation. Studies involving elements of stroke community support and an understanding of potential benefits or information about stroke are also desired.

**Conclusion:**

Collaborative research design can contribute to future research that is authentic and valuable to people with lived experience while concurrently lessening harmful disability narratives that are often unintentionally maintained by non‐disabled researchers. By working with a group of adults with chronic stroke, we were able to identify valued study features that can be incorporated into future stroke research studies.

**Lived Experience or Public Contribution:**

Individuals with lived experience of stroke engaged in the discussions presented in this paper to collaboratively identify desired stroke study features. People with stroke were also involved in the developing interpretation of the presented data and reviewed the final version of the manuscript.

## 1 Introduction

An estimated 9.4 million American adults self‐report having had a stroke. By 2030, it is projected that an additional 3.4 million adults will have had a stroke in the United States [1]. Stroke is a leading cause of long‐term disability, and full recovery is achieved only among a small number of stroke survivors [2]. Most people post‐stroke have residual impairments, such as weakness, tone, memory loss, and aphasia. Over 30% of people with stroke report significant participation restrictions, even after 4 years [3]. For many individuals and their families/caregivers, stroke is a profoundly life‐changing event [4, 5], often accompanied by feelings of grief and loss [6].

Participation in physical and occupational therapy is known to generally enhance physical and social function after stroke [4, 7]. The exponentially growing evidence base for stroke rehabilitation is a major factor in this clinical progress [8, 9]. Recent stroke rehabilitation research has made advancements in understanding post‐stroke gait [10, 11, 12, 13], postural control/balance [14, 15, 16], upper‐limb/hand function [17, 18], and community and social participation [19]. However, most stroke research studies do not compare researcher priorities with the priorities of research participants [20], as people with disability are often not meaningfully included in studies beyond acting as participants during data collection [21].

The field of rehabilitation has been informed largely by positivist assumptions—researchers present their own hypothesis about a set of variables and design a study to empirically test the relationship between those variables [22, 23]. Biomedical experts (e.g., scientists, funders) determine the disability narrative and make decisions about what studies should focus on rather than those most affected by the outcomes of research [21, 24]. Disability stands as a passive object of intellectual enquiry, and disabled voices and their pragmatic insights are notably absent [24, 25]. As such, researchers—specifically, non‐disabled researchers—may ask the wrong questions and/or introduce bias in the research and dissemination process [20, 26].

When research is not informed by the lived experiences of people with disability, studies are often perceived as irrelevant or absent of meaningful benefit [21, 27]. Further, many rehabilitation studies are complicit in subscribing to normative assumptions. For example, with regard to research about movement, the movement of non‐disabled people is considered the ‘ideal reference,’ and the movement of people with disability is considered a deviation from the ‘norm’ and an individualised problem to ‘fix.’ [24, 27, 28, 29] This functionalist perspective (even if unintentional) has been characterised by people with disability as ableist [5, 28, 30] which perpetuates the divide between researchers and people with disability. People with disability have criticised scientists for appearing to support their own professional interests rather than seeking to empower those with whom they work [31]. The historically limited involvement of people with stroke in physical and occupational therapy research fails to include people with disability in all aspects of life and give them a voice in matters that directly affect them [32]. These practices are inconsistent with the disability rights mantra, ‘Nothing About Us Without Us’ [32].

As research continues to evolve in stroke rehabilitation, it is critical that people with stroke are meaningfully involved in making decisions about all aspects of research—from priority setting to dissemination. There are multiple areas where people with lived disability experience can get involved in research: priority setting, research question development, grant writing, recruitment/collection, data analysis, and dissemination/implementation [33]. Comprehensive involvement of those with disabilities in research may help future research be more authentic and valuable to people with lived experience [34] while concurrently lessening harmful disability narratives that are often unintentionally maintained by non‐disabled researchers. Increased involvement from people with disability in research may also facilitate physical and occupational therapist attempts to be less ableist and promote an anti‐ableist workforce [27, 35, 36]. The purpose of the current study was to understand the key features and characteristics of physical and occupational therapy research studies desired by people with chronic stroke. This was our first step to inform a later co‐design phase aimed at collaboratively building original physical and occupational therapy studies with people post‐stroke. Our goal was to first identify desired study features via iterative focus group discussions and thematic analysis to facilitate the later co‐design of meaningful rehabilitation research studies for people with chronic stroke. This initial step further allowed us to provide a list of desired features for other stroke rehabilitation researchers to prioritise in their future studies.

### 1.1 Position Statement

The authors represent different identities and backgrounds, and we acknowledge that these different identities can influence our approach to science [37]. We are rehabilitation researchers currently with and without disability. We understand that there are limits to our experiences. We have academic backgrounds in physical therapy, occupational therapy, health sciences, disability studies, and psychology. We are collectively allies to the disability community, and we also recognise that there are times where we have inescapably perpetuated ableism and upheld barriers for the disability community. These identities inherently shape our presentation of results and interpretation of the current study.

## 2 Materials and Methods

### 2.1 Study Overview

Over a 4‐month period (September 2025 – December 2025), monthly iterative focus group discussions were held at an urban, public university in the midwestern United States or via secured virtual platform (i.e., Zoom) with the same group of individuals with chronic stroke. Each discussion emphasised a particular topic area to establish desired stroke rehabilitation study features from people with lived experience. These discussions were an exploratory step to inform a later planned co‐design project involving collaborative design and production of original physical and occupational therapy studies for people with chronic stroke.

Ethical approval was obtained by the University of Cincinnati Institutional Review Board (IRB), and all participants provided written informed consent at each session. We used the Consolidated Criteria for Reporting Qualitative Research [38] to guide our reporting and to ensure the methodological rigour of the study. We also referenced Braun and Clarke's recommendations for producing and reporting methodologically coherent thematic analysis [39].

### 2.2 Participants and Recruitment

Our goal was to recruit 4–6 individuals with chronic stroke to participate in all four focus group discussions. This sample size broadly falls within the recommended sample size for focus groups [40]. We intentionally kept the group size small to better manage conversation, maintain active engagement, and foster comfort with each other and with the research team. Smaller samples are ideal for more specialised topics and in‐depth conversation, allowing ample speaking time for all participants. We also wanted to facilitate retention of participants across all four discussions. Participants were eligible to participate if they were over 18 years of age and had a history of stroke > 6 months prior. Participants were excluded if significant cognitive, communication, or behavioural impairments precluded participation in discussions.

We purposively sampled from an existing stroke database used widely by stroke researchers at the university and the affiliated academic health centre. Participants were selected based on previous experience and participation in stroke studies, as the goal was to probe key features and characteristics of physical and occupational therapy research studies desired by people with chronic stroke. Therefore, it was imperative that participants had prior research experience so that they could leverage those experiences and give in‐depth input and feedback on research processes and methods. We also aimed to sample for a diverse range of stroke‐related impairments (e.g., upper‐limb, speech, balance, gait).

Potential participants were initially contacted by the first author, via email or telephone, to gauge interest and explain project goals. The final group consisted of five individuals with chronic stroke. On average, each member of the group participated in eight documented prior research studies at the university, across a variety of rehabilitation topic areas (e.g., gait, upper‐limb, balance, speech) and methods/instrumentation (e.g., virtual reality, motion capture, clinical trials). We anticipate the actual number of prior studies to be higher because many individuals had participated in opportunities outside of the university as well as opportunities that may not have been documented in the database. No participants dropped out of the focus groups; however, some participants could not attend every focus group discussion due to personal reasons, such as illness. Each participant missed one discussion, on average. No participants required communication support or adjustments. Participants were compensated for time and travel after each focus group discussion. Table 1 shows complete demographic information for the group.

**Table 1:** Group demographic information.

	Cohort (*n* = 5)
Age, y (mean ± SD)	62.0 ± 4.3
Gender, *n* (%)	
Female	2 (40.0)
Male	3 (60.0)
Race, *n* (%)	
African American/Black	2 (40.0)
Caucasian	3 (60.0)
Stroke chronicity, mo. (mean ± SD)	97.6 ± 25.8
Stroke type, *n* (%)	
Ischaemic	4 (80.0)
Haemorrhagic	1 (20.0)
Paretic side, *n* (%)	
Left	3 (60.0)
Right	2 (40.0)
Hours/week in physical or occupational therapy, *n* (%)	
No therapy	4 (80.0)
< 1 h	1 (20.0)
Highest level of education, *n* (%)	
High‐school diploma	2 (40.0)
Bachelor's degree	1 (20.0)
Graduate degree	2 (40.0)
Median annual household income,	
*n* (%)	
< $50,000	2 (40.0)
> $50,000	2 (40.0)
Prefer not to say	1 (20.0)
Stroke‐related communication disorder (*n*)	Aphasia (2), speech difficulty (1)
Device usage for mobility (*n*)	AFO + cane (2), cane (1), scooter (1), none (1)

### 2.3 Focus Group Discussions

Three focus group discussions were held in a conference room at the university with participants seated around a table with members of the research team. The final discussion was held via a secured virtual platform (Zoom) due to winter weather/travel concerns. Each discussion lasted 60–90 min. Some participants were familiar with each other from participation in prior stoke studies or from community stroke support groups. While these prior relationships were unknown to the research team at the time of recruitment, they were viewed favourably as it fostered a greater sense of community and comfort for participants. The first author was known to four of the five participants from prior research involvement.

At least three members of the study team were present at each of the four focus group discussions; the exact combination of study team members differed for each discussion session (based on scheduling availability). Session dates and times were decided collaboratively between the group and the research team at the end of the prior month's session.

Two authors (JF, KF) each moderated one session, and the first author moderated two sessions. Moderator academic backgrounds were in physical therapy (SMS‐F, JF) and occupational therapy (KF); all moderators identified as women. All moderators had graduate‐level research methods and statical training, including quantitative, qualitative, and mixed methods. To ensure consistency of moderating approach, the entire research team met prior to each focus group discussion to prepare for the upcoming topic. The team further debriefed after each session. A previous moderator was always present at discussions to intervene with additional probes or follow‐ups, further aiding in consistency.

Two of the authors (SMS‐F, VM) developed a focus group guide. The final guide was edited and approved by the study team 3 months prior to the commencement of focus group discussions. Each discussion focused on a specific thematic topic area, designed to gradually build to the final discussion topic focused on future topics for stroke rehabilitation research. Table 2 presents a summary of each focus group discussion with example probes. The full focus group discussion guide is available in Supplement 1. Discussions were iterative in the sense that they progressed from broad to more specific topics over each session and prior sessions could be referenced/layered upon. For instance, the first session focused on general thoughts on rehabilitation research. The second session honed in on specific prior research experiences. Participants were thus able to refer to discussions from the first session when discussing specific research experiences they liked/disliked in the second session, which allowed them to refine their view. There was opportunity at the start of each new session to return to ‘lingering thoughts’ from prior sessions in the event a participant changed their view or had additional thoughts on a topic. This strategy also helped account for missed sessions; participants who missed the prior session had the opportunity to provide input on the previous topic. All sessions were audio recorded for later analysis.

**Table 2:** Focus group discussion topics and example probes.

**Discussion Session 1: Thoughts on rehabilitation research** – Do you think more rehabilitation research focused on stroke is needed? – Do you feel that there are gaps in rehabilitation research related to stroke? – What are ways to encourage adults with stroke to participate in research?
**Discussion Session 2: Prior research experiences and movement after stroke** – What studies have you enjoyed in the past? Can you describe those studies? – What studies have you not enjoyed as much? Can you describe those studies? – What are aspects that you liked or did not like? – Did the research feel relevant and meaningful to you? – What movements are most meaningful to you?
**Discussion Session 3: The disability experience** – What are common assumptions that people make about your disability? – What should people without disability understand about your disability? – Do researchers design studies that feel meaningful and relevant to you?
**Discussion Session 4: The future of stroke research** – How can rehabilitation research studies be more relevant to people with stroke? – What are research priorities for you?

### 2.4 Data Analysis

Codebook thematic analysis [41] was used to analyse focus group discussions and identify desired features of rehabilitation studies for people with chronic stroke. Braun and Clarke view thematic analysis as a flexible process, wherein there is *plurality* to approaches rather than a singular method [39, 41]. There is a spectrum for thematic analysis, with coding reliability approaches at the positivist/post‐positivist end of the spectrum, and reflexive thematic analysis at the other; codebook thematic analysis falls somewhere between the two [41]. Codebook approaches reflect a pragmatic compromise in qualitative research. In codebook thematic analysis, a more structured approach to coding is taken through the use of a codebook and themes are developed using coding reliability approaches. Values of reflexive thematic analysis (e.g., researcher subjectivity) are also present [41]. The themes developed in the current study were intended to capture a core idea or meaning, rather than summarise a topic. This type of theme facilitates bringing together connected data, even between seemingly unrelated topics if a core idea is present [39, 41]. Instead of identifying themes *a priori*, we used an inductive approach to understanding the data.

All authors involved in data analysis were physical or occupational therapists with experience in stroke rehabilitation and research, and one of the authors involved in data analysis was not involved in data collection (DP) to enhance trustworthiness and reduce bias. First, recorded audio from the focus groups was transcribed verbatim and uploaded into the qualitative software Dedoose (v.10.0.59). Initial coding of transcripts was conducted independently by two of the researchers (SMS‐F, JF, or KF) to identify emergent groupings. Once independent coding was complete, the two coders met to collaboratively go over their initial coding of the transcript, line‐by‐line, compare coding notes, and establish a codebook. Any instances of differences were discussed until consensus was reached. Independent coder differences were viewed as opportunities for reflection and interpretative depth, not merely for consensus [41]. Through this iterative process, we re‐calibrated the definitions and applications of codes until the codebook was finalised (Supplement 2). A third coder (DP) then went through each coded transcript with the established codebook for a final review. Any differences were again resolved through discussion. After this phase, we reviewed and summarised quotations to abstract themes and categorised quotations exemplifying each theme by all members of the research team. The team developed, refined, and verified final themes through an iterative process and reflections.

To enhance trustworthiness and credibility of our analysis, study team members involved in data analysis regularly met to discuss codes and emerging themes as well as to discuss potentially alternative meanings. Team members maintained documentation of steps taken during analysis [42] for discussion at team meetings and to ensure that a similar process was completed by all coders. Additional steps to increase reliability included the use of field notes during collection and analysis. Independent coding was another step taken to enhance reliability and to support replicability of methods [41]. Finally, we share quotes in the presentation of our results to facilitate transparency and support our generation of themes [43].

Throughout our data analysis process, we provided monthly updates to the stroke group with preliminary findings (e.g., brief summaries of themes), and they were invited to provide comments, including disagreements, on the developing interpretation. The full draft of the results section and manuscript was later shared with all members the stroke group for feedback and comments. Four participants from the group provided feedback and agreed with the final themes; one participant did not respond to requests for feedback. Two of the participants provided feedback which required a revision of quote interpretation and further description of the theme (Theme 4) for clarity. These two individuals also provided several considerations and potential limitations that we included in the Discussion.

## 3 Results

Four major themes were constructed, each tied to a desired research study feature: (1) Desire for relationships; (2) Understand the purpose; (3) Capability‐oriented; and (4) Focused on individual participation needs. Each of these themes is detailed below with supportive quotes.

### 3.1 Theme 1: Desire for Relationships

*Subtheme 1a: Peer relationships*. The most recurrent idea shared by all members of the group across all discussion sessions was the value placed on stroke community support and the desire to integrate these sorts of relationships into research experiences.

Participants expressed how stroke can often be accompanied by isolation, loneliness, and depression. Sharing their personal story and resources with other stroke survivors can help mitigate those feelings, as shared by one participant:

…It's a scary situation to not have anybody when you have a stroke…If it wasn't for [support groups], I think I would be depressed.

(P5)

Relatedly, other participants indicated that prior relationships with friends and family are often lost or changed after stroke due to their limited understanding about the stroke experience, further contributing to the isolation experienced:

I think people who have not had a stroke or any major health concerns or conditions don't understand for the most part—when it comes to the general population, or when it comes to your family, even friends, that you had before the stroke…I lost a lot of people after I had a stroke.

(P3)

Because of relationship losses and isolation, participants indicated that the shared bond with other stroke survivors is highly regarded, even in the context of research participation. Participants noted how the stroke community brings experiences and resources that friends/family, healthcare providers, and researchers lack, repeatedly mentioning the value of support groups and each other:

I've been through a lot of studies, and… I listened to them. But the main thing that helped me was the stroke support meetings.

(P5)

I'm sure you've experienced how we share with each other and are sharing experiences with each other.

(P4)

Participants further described how support from other stroke survivors can translate to more favourable research experiences, noting this component as a desired study feature, as described by one participant:

Anything where we're sitting and talking, you know, and we know and feel that what each other are feeling, because we've experienced it, goes a long, long way, at least in my opinion.

(P4)

*Subtheme 1b: Researcher relationships*. In addition to support from fellow stroke survivors, a related recurring idea involved building relationships with researchers, with participants noting more favourable prior research experiences when there was opportunity to interact with members of the research team. Participants described that these relationships were more readily developed over multiple session studies, compared to single session studies, as noted by one participant:

Am I just cattle that's, you know, get weighed, measured, and off you go? And [in] the longer studies, I feel like I bought in more. Probably more gradual, but especially getting to know the [physical therapists] and the people, and you know, you go in every week for 4 or 8 or 12 weeks, and it becomes a much better experience, and you get more involved in the study itself.

(P1)

The same participant went on to describe more unfavourable research experiences that can occur from not having longer‐term interactions with researchers, particularly a feeling of limited involvement in the researcher's goals:

So, in some of the smaller [studies], I've expected to be included less or to be less informed…they're gathering data, they're not inviting me to participate in their goals.

(P1)

In summary, this theme captured a strong desire for an enhanced social component in physical and occupational therapy research studies, involving either increased engagement with other stroke survivors or members of the research team. Participants shared how stroke is a profoundly life‐changing event, often resulting in feelings of isolation and a loss of prior relationships. Being able to share experiences and build new relationships through research is highly prioritised.

### 3.2 Theme 2: Understand the Purpose

All participants discussed the desire for researchers to increase information related to study objectives and expectations, indicating that they are motivated to participate in studies when they understand the potential benefits, either for themselves or for others. One participant shared the common experience of feeling uninformed about study expectations in prior research experiences:

…a lot of times, we don't know what [to expect] …other than a stroke study.

(P3)

The same participant went on to explain why clarifying study objectives and providing better explanations for physical and occupational therapy studies can be motivating for participants:

I want to find out beforehand what I could expect after because, for me, the studies are just not getting the incentives. I want to get something from it. I mean, I want to be able to talk better, hear better, walk better, whatever.

(P3)

All participants shared the common sentiment that even if a study has no direct benefit to them, they still want to understand how it could potentially help other stroke survivors, as that too is motivating. One participant shared their specific motivation for participating in prior physical and occupational therapy studies—frequently stating that they did not experience any benefit through prior participation, but they continued to participate to see if they could attain additional information related to their recovery and for other stroke survivors:

So, the reason I did them was for two reasons, I guess. One was to find out other things I could do to help myself recover…And the second thing was, I felt like if what I do can help other people, then that's a benefit also.

(P2)

I think we all want something good to happen, and we can, you know, help everybody. I mean, that's why I do them, because…you know, if somebody else benefits…I mean, I'm all in.

(P2)

Another participant suggested that researchers increase information by providing study participants with results and further relating those aggregate results to individual performance:

Just that follow‐up with, here were the findings of the study, and this was what we would recommend for improvement of some sort.

(P4)

Finally, all participants agreed that when researchers provide additional information about study goals and objectives, it can increase hope for the future of stroke research studies:

I think these types of things give us hope. And you're looking into this and researching that there might be things coming down the pipeline that can help us.

(P4)

This theme supports the idea that physical and occupational therapy researchers should provide more information about study goals and objectives to people with stroke, and increased information is a desired study feature. Individuals in the group noted that an improved understanding of researcher goals and potential benefits/hypotheses can facilitate buy‐in and increase motivation for continued research participation.

### 3.3 Theme 3: Capability‐Oriented

The group expressed throughout discussions that they are constantly being told and reminded in their daily life of everything they cannot do by healthcare providers, family, and strangers. Disability is viewed as a problem to solve and something to ‘fix.’ These attitudes focused on ‘fixing’ individual deficits (e.g., body structure/function impairments) carry over to physical and occupational therapy research studies as well, and it can be a source of frustration, as shared by one participant:

That feeling of—and I'm not sure ego is actually the right word—but we all have a spot of frustration and maximum irritation…It's all too easy for a stroke study to inadvertently poke that spot…I absolutely hate hand studies. I can't move my hand.

(P1)

There is a desire for physical and occupational therapy research studies to increase emphasis on what people with chronic stroke can do, rather than what they cannot do. Participants indicated that they want to see studies focus more on their current situation, rather than deficits:

The picture I see is people trying to recover…and cope with the things they got going on in their post‐stroke life. And I think that's more important than just saying that, you know, 30% of these people couldn't use their right hand.

(P2)

Participants further shared that deficit‐focused research can feel unsatisfying, as expressed by one participant describing their involvement in a previous walking study:

…after 12 weeks of doing what [the researcher] told me to do, I never felt like I gained a ton. I mean, sure, I'm in better shape. I proved to myself that I could do something more quickly than, you know… I walked faster at the end than I did at the beginning, but—and it may be just a mental thing—but there wasn't a great feeling of accomplishment.

(P1)

Another participant provided additional insight into why deficit‐focused studies can feel unsatisfying, suggesting that ‘difference’ may sometimes reflect necessary adaptation after stroke, not something that needs to be ‘fixed’:

Because this is what I have to deal with… we have to reinvent ourselves. We have to find different ways to deal with certain things.

(P3)

The group also described prior studies that they enjoyed, in large part because they did not focus on specific deficits. Instead, they were told to ‘do what you can.’ All participants favourably commented on a prior mindfulness study as an example, as summarised by one participant:

That's why I absolutely love [researcher's] mindfulness study. Because we're all stroke. And we all can appreciate and know how each other are doing.

(P4)

In sum, this theme supports the development of studies that de‐emphasise individual deficits for people with chronic stroke, instead focusing on opportunities for success, creative movement solutions, and functionality. Participants noted how most physical and occupational therapy studies for chronic stroke are heavily deficit‐focused, serving as constant reminders of what they cannot do. Participants favoured prior research experiences that decreased focus on deficits and focused more on current capabilities.

### 3.4 Theme 4: Focused on Individual Participation Needs

The idea that ‘every stroke is different’ was prevalent across discussions. The failure to recognise the heterogeneity of stroke presentation was a major critique of researchers identified by the group. One participant expressed that they were unable to see the relevance of many physical and occupational therapy studies for stroke because, from their perspective, individual variability is often unaccounted for:

When I see these research studies, the fact that they're all so different, I'm not sure how I see that the data is really, you know relevant, I guess, because everybody's struggling so different, I'm not sure how really valuable the information is.

(P2)

Another participant shared a similar thought with respect to physical and occupational therapy research studies, indicating that even when there are strict inclusion/exclusion criteria, the research remains limited because of individual stroke variation:

So then, when you get a stroke survivor, and everybody is different, my stroke is different from theirs, and theirs are different from mine… It's a whole new loophole, because you [have] three different people who had strokes, but all of our deficits are not the same.

(P3)

Relatedly, there was a general feeling that some inclusion/exclusion criteria for stroke research studies seem random, irrelevant, and perhaps too stringent which may limit who can participate and reduce the amount of available data, as indicated by one participant:

If you can't let your pinky touch your chin three times, you can't participate.

(P1)

The group devised a solution to account for individual variation in stroke presentation in the final focus group discussion, consistent with individualised outcome measures. They suggested broader inclusion criteria and implementing an approach centred on individual goals:

…You could have four people together, and we have a common task. And in the end, after it's completed, you see how they benefited, or they improved on something. So, like, for me, with my speech, you know, with interacting with people, that I may realise a benefit from my speech improving, because I participated in that. Um, or somebody that can't use their right hand, maybe they found ways to use [their] right hand in different ways, or whatever.

(P2)

Overall, this theme captured that people with chronic stroke recognise the individual nature of stroke presentation—a recognition that is sometimes felt by people with stroke to be absent among researchers. To account for this variability, study features involving individual outcome measures and broader inclusion criteria are desired.

## 4 Discussion

This study aimed to understand desired physical and occupational therapy research study features and characteristics for people with chronic stroke. We identified four major study features: (1) Desire for relationships; (2) Understand the purpose; (3) Capability‐oriented; and (4) Focused on individual participation needs. These features will be used to inform our next phase of co‐design and can be more widely used by other stroke rehabilitation researchers to potentially enhance the relevance of their studies.

### 4.1 Desire for Relationships

The strongest idea that emerged from our discussions was related to the value placed on community support and forming new relationships after stroke. Many chronic conditions, including stroke, can bring about feelings of isolation and loneliness—a sentiment frequently expressed by members of our group. Interactions with people who have shared experiences can provide a unique sense of community, belonging, and unconditional acceptance, as opposed to the lack of knowledge and rejection often encountered from interactions with people without those shared experiences (e.g., family, friends, healthcare providers, researchers) [44, 45, 46]. Prior research supports this notion of a desire for relationships, finding that people with stroke prioritise the development of a therapeutic alliance or relational approach from their therapists in clinical settings [47] and place value on personal connections [48].

This theme recognises that stroke involves not only physical changes, but complex and context‐dependent psychological challenges as well [49]. Individuals post‐stroke are tasked with accepting current life changes and engaging in new roles/activities [5, 49]. While individuals post‐stroke may find their current life meaningful, it may still be accompanied by feelings of grief and loss [5, 6]. Social support has been found to be important for post‐stroke adjustment [49], and the results of the current study point to social support as something highly desired in physical and occupational therapy research studies as well—incorporated either in study design or as the subject matter. It is also important to consider the role played by stroke researchers in stroke survivors’ new social network and how the relationships formed with members of the research team can shape their perception of recovery. Integrating study features that can foster those new relationships (e.g., multiple session designs, collaborators with stroke) is relevant for stroke rehabilitation researchers to reflect upon for future work, recognising potential trade‐offs and limitations as well. For instance, multiple session studies may increase engagement but also introduce risk of attrition. Further, simply increasing the amount of time spent with a research team may not directly translate to quality and meaningful relationships.

### 4.2 Understand the Purpose

Participants in the current study indicated a desire for physical and occupational therapy researchers to augment available information related to potential study benefits (for self or others), hypotheses, and study objectives. Participants voiced that a clearer understanding of studies can give them something to positively anticipate, help them appreciate study relevance, and enhance motivation to participate, stating that, oftentimes, they only know that they are participating in a stroke study without full awareness of study goals. This desired feature is consistent with an increasing volume of literature across multiple health research domains which indicates that additional information about research studies is prioritised, and this has been voiced especially among minoritized groups where there is a history of uninformed participants [50, 51, 52, 53, 54]. Overall, this theme highlights concerns regarding informed consent to ensure that research participants truly are well‐informed [55]. Furthermore, following‐up after the study with aggregate results as well as with individual results was suggested by participants in the current study as a way to additionally address study information and potential benefits.

It is important to note that, while this was a desired study feature, researchers should be aware of potential ethical issues that could arise in their own work related to potential benefit. Research participation will not always lead to personal improvement or benefit, and there is a risk of misconception if individuals believe that a study was designed to directly benefit them, rather than answer a basic research question. Together, this ethical concern reiterates the need for transparency in the information provided about research and further emphasises the need to ensure informed participants.

### 4.3 Capability‐Oriented

The desire for capability‐oriented studies by individuals with chronic stroke reflects the continued dominance of medical models underpinning physical and occupational therapy research studies, despite calls for the implementation of more relational models of disability [27, 30, 56, 57, 58, 59]. Medical models of disability assume linear casual logic from (1) structural impairment (i.e., deficit) to (2) performance limitations to (3) subsequent disability. Thus, ‘fixing’ a deficit assumes that ‘normalisation’ will provide the best path to function, participation, and independence [28, 29, 30, 59]. Deficit frameworks—while producing focused data—fail to account for the broader sociopolitical, environmental, and economical contexts that also contribute to disability [27, 35, 56, 60, 61]. From a philosophical standpoint, it has been argued that the rehabilitation professions have adopted an ‘ontology of transcendence’—an ontology that posits the physical world is an imperfect reflection of a perfect realm [62]. Accordingly, ‘difference’ is understood as imperfection, and the rehabilitation professions are sustained by being able to locate and erase markers of difference (i.e., deficits) [62].

The skewing of the rehabilitation professions toward deficit frameworks is well‐established [27, 29, 35, 59, 63]. In fact, many post‐stroke guidelines view rehabilitation as an ‘expert‐driven, impairment‐focused biomedical procedure.’ [64] The harm of this positioning is that it contributes to health inequities experienced by people with disabilities [35] and negative rehabilitation experiences [27, 35]. Extensive emphasis on deficit—either by a therapist or researcher—can reinforce the negative self‐perceptions [65] that come from being defined by others only in terms of bodily attributes post‐stroke. [66], Participants in the current study noted that life is different after stroke, and they have to find new ways to ‘re‐invent’ themselves. Designing studies that embrace difference and focus on creative and adaptable movement solutions and functionality (rather than deficits) [5, 30, 58] may facilitate this re‐invention. Concurrently, shifting the guidelines informing clinical practice away from impairment‐focused toward re‐engagement in valued activities and roles [64, 67] may also help to achieve this vision and help to foster a more anti‐ableist workforce.

### 4.4 Focused on Individual Participation Needs

The heterogeneity of stroke presentation, and the related need to address this individual variation in research studies, was repeatedly brought forth by members of the chronic stroke group. Individualised outcome measures (e.g., Goal Attainment Scaling [68], the Canadian Occupational Performance Measure [69]) are often discussed and implemented in clinical rehabilitation contexts [70], but the results of the present study support their addition to the battery of outcome measures used in stroke physical and occupational therapy research studies as a step to increase study relevance and meaning. This approach recognises that every stroke is different, and thus, every research participant is going to want something different from their research participation.

Broadening inclusion criteria was also addressed as a desired study feature to embrace individual variation. Participants commonly felt like study inclusion/exclusion criteria were random and irrelevant, especially given that stroke presentation varies substantially, even when inclusion criteria are met. For instance, study samples may meet inclusion criteria in terms of functional deficits—and appear as a homogenous sample—but they still will inherently vary in terms of personal and contextual factors, which are known to influence movement outcomes [56]. This consideration regarding inclusion/exclusion criteria further underscores the need to address the previously discussed theme of de‐emphasising deficits in research. It is worth noting that individual, participation‐focused research—while desired by people with stroke—may be difficult to fund given the biomedical focus of many national and international stroke guidelines [64].

### 4.5 Future Work and Implications

Our next step is to work with individuals with chronic stroke to co‐design an original physical and occupational therapy research study that includes the features identified in this study. We intend to work with co‐researchers to plan, design, pilot, and disseminate the study.

There are several existing areas of stroke research in physical and occupational therapy that take advantage of the features identified in the current study. Photovoice [71, 72], mindfulness [73, 74, 75], and yoga [74, 76, 77] are several examples of research that incorporate features of stroke community, individual outcomes/individual variation, and capabilities over limitations. Table 3 presents specific examples of how stroke rehabilitation researchers can integrate desired study features into their studies. Stroke researchers can incorporate some of these examples into their research to enhance study desirability and relevance. For instance, if a stroke rehabilitation researcher focuses their research agenda on grip strength, they might not be able to create a fully capability‐oriented study, but they can still include a person with stroke on their research team and provide participants with a short report about their performance at the end of the session. Researchers can certainly include all features, but even incorporating 1–2 features into studies may make a difference in research experiences for people with chronic stroke.

**Table 3:** Examples of how to implement desired study features into research studies.

Desired study feature	Examples
Desire for relationships	Group studies that involve interacting with other people with stroke Include people with stroke on research team and have them involved with data collection Multiple session studies with the same members of the research team Involve community stroke support groups for feedback on studies being prepared
Understand the purpose	Increased time during informed consent process explaining study purpose Share hypotheses with participants during debrief period Share results of study at its conclusion and share individual data, if possible Include informational materials about stroke
Capability‐oriented	Allow multiple movement solutions to achieve study goal Provide opportunities for success Design study tasks that are not contingent on a specific impairment/deficit Recognise contextual factors contributing to disability
Focused on individual participation needs	Use of personalised outcome measure as part of assessment battery (e.g., GAS, COPM) Provide rationale for inclusion/exclusion criteria Determine inclusion/exclusion criteria with stroke co‐researchers

### 4.6 Limitations

The results of this study should be considered with respect to its limitations. The experiences shared may be specific to the small group in this study, from our specific university in the midwestern United States, and may not be representative of the global stroke community. While it was a strength that our sample had substantial prior research experience, people with stroke who have not participated in research previously may identify different desired study features. Additional sample limitations included: existing relationships with some researchers, existing relationships with other group members, possible volunteer bias, and the exclusion of people with greater cognitive or communication needs. It is also important to reiterate that members of the group were in the chronic phase post‐stroke. It is possible that individuals in more acute or subacute phases have different desired research study features, and those should be explored in future work.

## 5 Conclusion

Collaborative research design facilitates research that can be authentic and valuable to people with lived experience while concurrently lessening harmful disability narratives that are often unintentionally maintained by non‐disabled researchers. By working with a group of adults with chronic stroke, we initiated the collaborative research process by first identifying four valued study features that can be incorporated into future stroke research studies in physical and occupational therapy.

## Author Contributions

**Sarah M. Schwab‐Farrell:** conceptualisation, investigation, funding acquisition, writing – original draft, methodology, writing – review and editing, formal analysis, project administration, supervision, resources. **Jasmine Ficke:** formal analysis, investigation, writing – original draft, writing – review and editing. **Daria Pressler:** formal analysis, writing – review and editing. **Kelly Farrell:** formal analysis, investigation, writing – review and editing. **Becca Rose:** investigation, writing – review and editing, formal analysis. **Valerie Miller:** formal analysis, investigation, methodology, supervision, writing – review and editing.

## Ethics Statement

All participants gave written informed consent. The study was reviewed and approved by the University of Cincinnati Institutional Review Board (IRB).

## Conflicts of Interest

The authors declare no conflicts of interest.

## Supporting information


Supporting File 1

Supporting File 2

## Data Availability

Data available upon reasonable request to the corresponding author.
