# Supplementary material for: ‘Some of Them Were Created by People Who Have Never Had a Stroke’: Understanding Desired Physical and Occupational Therapy Research Study Features for People With Chronic Stroke
Source: Health Expect. 2026 Oct 8;29(5):e70918. doi: 10.1111/hex.70918 (PMC13648021; doi:10.1111/hex.70918)
Supplement: Supplementary file 1 [file HEX-29-e70918-s001.docx]

**Supplement 2: Coding Manual**

**Barriers to participating in research** – statements regarding barriers to research participation

- Accessibility barriers – transportation, location; mention of a study being inaccessible or inconvenient
- Emotional barriers – frustration or ego interfering with research task
- Inclusion/exclusion criteria – perceived exclusion/inclusion criteria of a study as a barrier to participation

**Comments on life after stroke** – statements that give an impression or describe life after having a stroke

- Ableism after stroke – comments that suggest ableism by society, friends, family, or medical professional after stroke
  - Abandonment – description of abandonment by society, friends, family, or medical professional after stroke
  - Perceived lack of stroke knowledge/understanding from others – statement gives an impression that those without stroke do not understand those who have had a stroke
- Deficits after stroke – description of a deficit or impairment experienced after stroke
- Information after having a stroke – encompasses a lack of resources offered (or rather not offered) after someone had a stroke; also includes limited information provided to family and friends resulting in consequences (e.g., loss of relationships) that impacted the stroke survivor’s life
- Stroke story – description of personal stroke experience/events leading up to stroke (i.e., origin of stroke)
- Positive perception of recovery – statements related to participants feeling like they recovered well after stroke

**Concurring with previous statements** – explicit agreement with other participants or a statement the interviewer made

**Continuous visits** – discussion of multiple sessions for a research study

- Positive perception of structure – gives impression that participant enjoyed the structure of multiple session studies

**Critique of researcher perspective** – statements that indicate a disconnect between the researcher and participants

- Research is deficit-focused – statements that previous studies focused heavily (perhaps too heavily) on a deficit, making the participant feel frustrated

**Doubting own experience** – statement indicates they are unsure of their own experience

**Interactions with researchers** – mentions a researcher from a previous research experience or indications of enjoyment from having opportunity to speak with/work with researcher

**Location of research studies** – literal description of where a participant had to go for a research study

**Modification of activity to accommodate all participants** – description of a research activity that was modified or discussion of day-to-day modifications of activity

**Motivation to participate in research** – statements giving reasons/impression of what motivates participants to be involved in research studies

- Closing insurance gap – motivation to participate in studies to get continued therapy due to insurance limitations
- General perception of benefits to participating – general statement expressing willingness to participate
- Helping the researcher – helping researcher achieve their goals is a factor in participation
- Helping other stroke survivors – helping other stroke survivors is a factor in participation
- Hope for the future of stroke – statement expressing motivation to participate because it provides a source of hope for the future
- Improved understanding of researcher objectives –statement gives the impression that more knowledge regarding a study would be motivating by providing an example of when that would happen or an explicit statement of such
- Self-improvement – reason for participation is for self-improvement and personal gains

**Prior research experiences** – literal description of previous research experiences

- Continued participation despite doubts – statements expressing a desire to continue to participate despite not seeing the benefit for themselves or in general
- Lack of perceived gain – statements expressing not gaining anything from previous research experience
- Physical improvement despite low perceived benefit – mention of physical improvement with impression of not really gaining anything meaningful from study
- Lack of understanding study purpose – literal statements or implied statements of not understanding the reasoning behind a study that they previously participated in
- Perceived lack of difficulty in research task – description of research task not being challenging enough while also giving impression that the task was not beneficial either
- Perceived randomness of task – perceived meaninglessness of prior research task
- Positive perception of research task – general statement that gives impression of enjoying an activity from a previous research study
- Specific activities completed in prior research studies – description of literal activities/tasks that the participant has previously done in other research studies
- Timeline of participation – literal description of when participation in research studies began
- Wide variety of research experiences – statements indicating breadth of research experiences

**Recognition of individual variability** – statements indicating that every stroke is different

- Individual variability as a perceived barrier – statements indicating that every stroke is different and indicating that this is a perceived barrier to effective research

**Recruitment** – description of method of recruitment or some other related aspect of recruitment for a research study

**Singular visits** – description of cross-sectional study design or single visit designs

**Stroke community or stroke support** – statements related to support groups or accessing support as a stroke survivor

- Stroke community as a resource – using stroke community to gain resources (e.g., information on services clinicians, etc.)
