# Supplementary material for: ‘Some of Them Were Created by People Who Have Never Had a Stroke’: Understanding Desired Physical and Occupational Therapy Research Study Features for People With Chronic Stroke
Source: Health Expect. 2026 Oct 8;29(5):e70918. doi: 10.1111/hex.70918 (PMC13648021; doi:10.1111/hex.70918)
Supplement: Supplementary file 2 [file HEX-29-e70918-s002.docx]

**Supplement 1: Focus Group Discussion Guide**

**Introduction:** Thank you for taking the time to participate today. Your input is very important and will contribute to the development of future stroke research studies that are relevant, authentic, and meaningful to people with stroke. During this discussion, we will talk about your thoughts on rehabilitation research, prior research experiences, and what you would like to see rehabilitation (physical therapy, occupational therapy) researchers focus on. If there are any questions you would prefer not to answer, please let me know.

**Recording:** I would like to record this discussion for note taking purposes. You do not have to agree to be taped; you can still participate in this conversation if you do not want to be taped. Please let me know if you agree to be recorded.

**Discussion 1:** Tell me your thoughts on rehabilitation research. These are studies that are focused on physical or occupational therapy. What comes to mind when you think about rehabilitation research studies?

- Potential Probes:
  - What are benefits of research?
  - What are barriers to research?
  - Do you think rehabilitation research is needed? Why or why not? (Probe: What sort of value, if any, do clinical trials or studies provide?)
  - Do you think more rehabilitation research focused on stroke is needed? Why or why not?
  - Do you feel represented or included in current research studies?
  - Do you feel that there are gaps in rehabilitation research related to stroke?
  - What are ways to encourage adults with stroke to participate in research?

**Discussion 2:** Can you tell me about your prior research experiences?

- Potential Probes:
  - Is there any research that you have heard about specifically? (Probe: How did you hear about this research?)
  - Have you been asked to participate in research? (Probe: If so, what was that experience like? How did you feel about being asked to participate? Did you participate?)
  - If you haven’t participated in studies, what are some reasons why you haven’t participated or what are some reasons you would choose to not participate?
  - If you have participated in studies, what are the main reasons why you chose to participate?
  - What studies have you enjoyed in the past? Can you describe those studies?
  - What studies have you not enjoyed as much? Can you describe those studies?
  - What are aspects that you liked or did not like?
  - Did the research feel relevant and meaningful to you?

Can you tell me a little bit about how your body moves with stroke?

- Potential Probes:
  - What are activities that you would like to be able to do or be better at doing?
  - What movements are most meaningful to you?
  - Are there movements that you struggle with that you would like rehabilitation research to address?
  - Are there ways you would like to more fully participate in society?

**Discussion 3:** Can you tell me a little bit about your experience as a person with disability?

- Potential Probes:
  - What are common assumptions that people make about your disability?
    - Follow-up: Who makes these assumptions?
  - What should people without disability understand about your disability?
  - Do you feel like physical and occupational therapists understand your disability? Do physical and occupational therapists make assumptions about your disability?
  - Do you feel like researchers understand your disability? Do researchers make assumptions about your disability?
  - Do researchers design studies that feel meaningful and relevant to you?
  - Do you feel like research studies remain relevant to you as you age?
  - Do physical and occupational therapists create clinical sessions that are meaningful to you?
  - Do researchers design studies that are too easy/too challenging? Are the studies engaging?
  - Do physical and occupational therapists design treatment sessions at the appropriate level of challenge/engagement?
  - Do you feel a sense of belonging during participation in research?
  - Do you feel a sense of belonging during participation in physical or occupational therapy?
  - Do you feel like there are gaps in your knowledge related to your disability?
  - Do you feel like there are gaps in your physical therapy/occupational therapy providers’ knowledge related to your disability?

**Discussion 4:** What are your ideas for future research for stroke?

- Potential Probes:
  - How can rehabilitation research studies be more relevant to people with stroke?
  - What type of research would be most meaningful to you to participate in?
  - Are there any rehabilitation studies you would like to see?
  - How can research address changes that happen with age?
  - What are research benefits that you would like to see?
  - What are research priorities for you?
    - Probes: Do you think walking, balance, upper limb, vision, or mental health studies would be most impactful?
  - How can rehabilitation research be more inclusive of people with varying levels of disability?
